# Efficacy and Safety of Biologic and Targeted Synthetic DMARDs in Young-Onset Rheumatoid Arthritis: A Systematic Review

**DOI:** 10.3390/life16020225

**Published:** 2026-01-29

**Authors:** Mara Russu, Vladia Lăpuște, Diana Elena Cosău, Alexandra Lori Donica, Alexandra-Diana Diaconu, Georgiana Strugariu, Cristina Pomîrleanu, Codrina Ancuța

**Affiliations:** 1 Faculty of Medicine, Grigore T Popa University of Medicine and Pharmacy Iasi, 700115 Iasi, Romania; mara.russu@umfiasi.ro (M.R.); costachescu.alexandra-lori@d.umfiasi.ro (A.L.D.); daniela.pomirleanu@umfiasi.ro (C.P.); 2 2nd Rheumatology Department, Clinical Rehabilitation Hospital, 700661 Iasi, Romania; 3 2nd Internal Medicine Department, “Sf. Spiridon” Clincal Emergency Hospital, 70011 Iasi, Romania

**Keywords:** young-onset rheumatoid arthritis, YORA, biologic DMARDs, targeted synthetic DMARDs, TNF inhibitors, JAK inhibitors, IL-6 inhibitors, treatment persistence, early-adulthood RA

## Abstract

**Background**: Young-onset rheumatoid arthritis (YORA), defined by disease onset between 16–40 years, raises distinct clinical challenges related to long-term disease burden, fertility, and prolonged exposure to immunomodulatory therapy. Despite its relevance, evidence regarding treatment outcomes in this population remains limited and heterogeneous, largely due to inconsistent definitions of YORA across studies. **Methods**: This systematic review was conducted in accordance with the PRISMA 2020 guidelines to synthesize contemporary evidence on the efficacy and safety of biologic and targeted synthetic disease-modifying antirheumatic drugs (b/tsDMARDs) in younger rheumatoid arthritis populations. A structured search of PubMed and Embase was performed to identify studies published between 2020 and 2025 that evaluated advanced therapies in patients with young-onset rheumatoid arthritis or in rheumatoid arthritis cohorts reporting age-stratified outcomes for younger adults. **Results**: From the screened literature, 16 studies met the predefined inclusion criteria, including 6 studies explicitly defining YORA based on age at disease onset and 10 studies reporting outcomes in younger adult subgroups (<40–45 years). Across studies, younger patients demonstrated higher remission rates, greater reductions in disease activity, and superior treatment persistence compared with older-onset rheumatoid arthritis cohorts. Tumor necrosis factor inhibitors, interleukin-6 receptor antagonists, and Janus kinase inhibitors showed consistent clinical efficacy. Structural outcomes, reported in a limited number of studies, suggested low rates of radiographic progression in younger patients. Safety profiles were generally favorable, with infections and laboratory abnormalities representing the most reported adverse events and no age-specific safety signals being identified. **Conclusions**: Biologic and targeted therapies provide substantial clinical benefit in YORA and younger adult RA populations, with outcomes being generally superior to those observed in older-onset RA. However, heterogeneity in YORA definitions and limited long-term data highlight the need for prospective, age-at-onset-defined studies and extended pharmacovigilance to better inform lifelong treatment strategies.

## 1. Introduction

Rheumatoid arthritis (RA) is a chronic, immune-mediated inflammatory disease characterized by persistent synovitis, progressive structural damage, and substantial functional impairment [1,2,3,4]. Although its prevalence increases with age, RA may begin at any point during adulthood, and the age at disease onset represents a critical determinant of disease phenotype, long-term prognosis, and cumulative treatment burden [5,6,7]. Among the various onset subtypes, Young-Onset Rheumatoid Arthritis (YORA)—typically defined as onset between 16 and 40 years—constitutes a clinically distinct yet insufficiently characterized subgroup within the broader RA spectrum [8,9].

The onset of RA in early adulthood carries specific implications. Patients face a prolonged lifetime with the disease, including many decades of potential exposure to disease-modifying therapies [10,11,12]. Moreover, RA onset during early reproductive and socio-professional periods introduces unique challenges related to family planning, fertility considerations, educational trajectory, career development, and psychosocial impact [13,14,15,16]. Despite these features, YORA remains underrepresented in clinical research, as most randomized controlled trials and large registries report aggregated outcomes across broad age ranges, rarely stratifying by the age at disease onset [17,18,19].

Existing evidence suggests that younger adults may differ from older-onset RA patients in terms of immunopathology, comorbidity profile, and response to therapy [20,21,22]. Younger individuals often present with fewer comorbidities, potentially enabling earlier introduction and sustained use of biologic (bDMARDs) or targeted synthetic DMARDs (tsDMARDs) [23,24,25,26]. Preliminary data also indicate that younger patients may achieve higher remission rates and demonstrate superior treatment persistence, although these findings remain inconsistent due to substantial methodological variation across studies [27,28,29,30]. Definitions of YORA vary widely, ranging from strict onset-based criteria (16–40 years) to broader subgroup analyses using age at enrollment (<40 or <45 years), complicating cross-study comparability [31,32].

Importantly, chronological age at assessment is not equivalent to age at disease onset, as older cohorts may include individuals with long-standing young-onset disease. Furthermore, early rheumatoid arthritis defined by short disease duration represents a distinct clinical construct and should not be conflated with YORA. Failure to distinguish these entities complicates cross-study comparisons and obscures age-specific treatment trajectories.

Given these disparities, a dedicated synthesis of age-stratified evidence is necessary to clarify therapeutic outcomes in younger RA populations. While previous reviews have addressed early RA defined by disease duration and not age at onset, or elderly-onset disease (EORA), few have specifically addressed the implications of age at onset in the context of advanced therapies such as TNF inhibitors, IL-6 receptor antagonists, JAK inhibitors, abatacept, or rituximab [33,34,35,36] particularly in the context of contemporary biologic and targeted synthetic therapies. Understanding how younger adults respond to bDMARDs and tsDMARDs is essential for optimizing long-term treatment strategies, minimizing radiographic progression, and tailoring care to a patient population with distinctive life-stage needs [37,38,39].

The present systematic review aims to consolidate available data on the efficacy, radiographic outcomes, treatment persistence, and safety of biologic and targeted synthetic DMARDs in YORA and in RA cohorts reporting outcomes among younger adult subgroups. By integrating evidence from both explicit YORA studies and age-stratified analyses, this review provides an updated, age-focused perspective intended to inform clinical decision-making and highlight gaps requiring future investigation.

## 2. Materials and Methods

### 2.1. Study Design and Reporting Standards

This systematic review was conducted in accordance with the PRISMA 2020 guidelines. A predefined methodological framework, including the research question, eligibility criteria, search strategy, study selection procedures, and data extraction protocol, was established prior to initiating the review. The review was not registered in PROSPERO because no primary patient data were collected or analyzed.

### 2.2. Search Strategy

A focused literature search was performed in PubMed and Embase for articles published from January 2020 to November 2025, with the specific aim of identifying studies addressing young-onset rheumatoid arthritis (YORA). This timeframe was intentionally selected to capture contemporary real-world and clinical trial evidence from the biologic and Janus kinase inhibitor era, reflecting current treatment strategies, regulatory updates, and evolving safety profiles relevant to long-term management in younger patients.

The search strategy combined Medical Subject Headings (MeSH), Emtree terms, and free-text keywords related to age at onset and advanced RA therapies. Search terms included “young-onset rheumatoid arthritis”, “YORA”, “young adult rheumatoid arthritis”, “age at onset”, in combination with “biologic DMARDs”, “bDMARDs”, “targeted synthetic DMARDs”, “JAK inhibitors”, “TNF inhibitors”, and “IL-6 receptor antagonists”. Boolean operators (“AND”, “OR”) were used to optimize sensitivity and specificity. Only articles published in English were considered. Reference lists of all eligible full-texts were manually screened to identify additional relevant studies. The initial YORA-focused search retrieved 210 records across the two databases.

### 2.3. Eligibility Criteria

Eligibility was defined using the following criteria:

Population: adult patients with rheumatoid arthritis diagnosed according to accepted classification criteria, including the 2010 ACR/EULAR criteria. Patients were eligible if they met one of the following conditions: (a) young-onset RA, defined by disease onset at or before the age of 40 years; or (b) inclusion within RA cohorts that reported treatment outcomes separately for younger adult patients, typically defined by chronological age at enrollment (<40–45 years). Studies enrolling broader age ranges were included only when age-stratified data for younger adult patients were explicitly reported, allowing extraction of outcomes relevant to this population. Throughout the review, a clear distinction was maintained between age at disease onset and chronological age at study assessment, recognizing that these parameters are not clinically interchangeable.

Interventions: biologic DMARDs (TNF inhibitors, IL-6 receptor antagonists, abatacept, rituximab) or targeted synthetic DMARDs (JAK inhibitors).

Outcomes: at least one clinically relevant endpoint, including remission or response rates, radiographic progression, treatment persistence, or safety outcomes.

Study design: randomized controlled trials, prospective or retrospective cohort studies, registry-based analyses, or cross-sectional studies with extractable age-stratified data.

Exclusion criteria were studies evaluating early RA based solely on disease duration (<2 years) without reporting age at onset, cohorts restricted to elderly-onset RA (EORA), pediatric-only studies that did not transition into adult RA, narrative reviews, case reports, editorials, and articles lacking age-specific or clinically relevant outcomes.

### 2.4. Study Selection

After automatic and manual deduplication, 36 duplicate records were removed, leaving 174 unique studies for title and abstract screening. Of these, 99 records were excluded because they did not meet the eligibility criteria regarding population characteristics, study design, intervention type, or outcome relevance. A total of 75 full-text articles were assessed for eligibility and successfully retrieved.

Among these, 59 full-text articles were excluded for predefined reasons: wrong study population (*n* = 33), or lack of separate young-onset or younger adult subgroup data (*n* = 26).

Finally, 16 studies met all inclusion criteria and were included in the qualitative synthesis. Six studies explicitly defined young-onset rheumatoid arthritis (YORA) based on age at disease onset, while ten additional studies reported outcomes for younger adult subgroups (<40–45 years) and were therefore classified as borderline but eligible.

**Figure 1:**
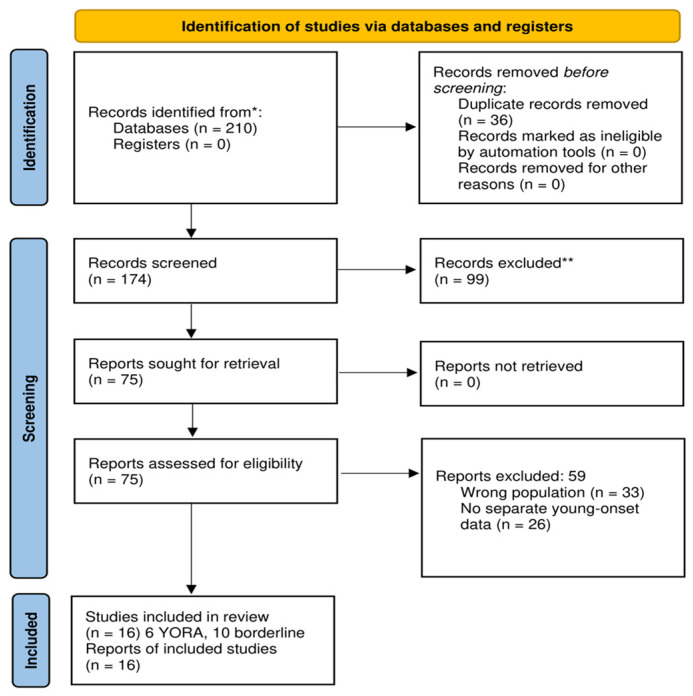
**PRISMA 2020 flow diagram of the study selection process.** *: Records were identified through database searching (PubMed and Embase); no records were identified through registers.

A PRISMA 2020 flow diagram summarizing the study selection process is presented in Figure 1.

### 2.5. Data Extraction and Synthesis

Data extraction was independently performed by two reviewers using a structured template. Extracted variables included: study design, country, sample size, demographic characteristics, definition of age at onset or subgroup, type of biologic or targeted therapy administered, remission and response rates, radiographic findings (if available), treatment persistence, and safety outcomes. Due to considerable heterogeneity across study methodologies, populations, and endpoints, the results were synthesized descriptively rather than through quantitative meta-analysis.

Given the substantial heterogeneity in study design, age definitions, outcome measures, and follow-up duration, quantitative meta-analysis was not feasible. Results were therefore synthesized descriptively, in line with PRISMA recommendations, to provide a structured qualitative and quantitative overview of available evidence.

### 2.6. Risk of Bias Assessment

The methodological quality of observational studies was evaluated using the Newcastle–Ottawa Scale (NOS), while randomized controlled trials were assessed using the Cochrane RoB 2.0 tool. Risk-of-bias assessments were used to contextualize the strength of evidence but did not serve as exclusion criteria. The predominantly observational and registry-based nature of the included studies was recognized as a potential source of bias, including confounding by indication and selection bias, and was considered when interpreting the findings.

## 3. Results

### 3.1. Overview of Included Studies

A total of 16 studies met the inclusion criteria and were included in the qualitative synthesis. Among these, 6 studies explicitly defined young-onset rheumatoid arthritis (YORA) using an age-at-onset threshold of 16–40 years, while 10 studies reported outcomes for younger adult RA subgroups (<40–45 years) and were therefore classified as borderline but relevant. Together, these studies represent the most consistent evidence addressing age-stratified treatment outcomes in adult RA populations.

The included studies comprised randomized controlled trials, prospective and retrospective cohort studies, and registry-based analyses. Most studies were observational or registry-based and therefore carried a moderate inherent risk of bias, which was considered when interpreting comparative effectiveness outcomes.

Sample sizes ranged from fewer than 100 to more than 5000 participants, and follow-up durations varied from 6 to 24 months, with only a few studies providing longer-term data. Studies with mean or median cohort ages exceeding 50 years were classified as providing age-stratified supportive evidence rather than representing true YORA populations.

Key characteristics of the included studies are summarized in Table 1.

### 3.2. Clinical Remission and Treatment Response

Across the included studies, younger patients—whether classified as YORA or as younger adult subgroups—consistently demonstrated higher clinical response and remission rates compared with older-onset RA populations. Reported DAS28 remission rates in YORA cohorts typically ranged from approximately 40% to 60% at 6–12 months of follow-up, particularly in studies evaluating early initiation of biologic or targeted synthetic DMARDs. Multiple registry analyses identified age as an independent predictor of treatment response, with younger patients achieving greater reductions in disease activity scores and higher proportions of Boolean remission. These trends were consistent across all drug classes (TNF inhibitors, IL-6 receptor antagonists, JAK inhibitors, abatacept), suggesting that improved outcomes reflect patient-related rather than drug-specific factors. Age-stratified registry analyses showed similar trends, with younger adults achieving greater reductions in disease activity scores and higher proportions of low disease activity or Boolean remission than older patients. Several studies identified younger age as an independent predictor of treatment response, irrespective of drug class. These findings were consistent across tumor necrosis factor inhibitors, interleukin-6 receptor antagonists, Janus kinase inhibitors, and abatacept, suggesting that favorable outcomes were driven primarily by patient-related factors rather than therapy-specific effects.

### 3.3. Radiographic Outcomes

Radiographic outcomes were reported in a limited subset of studies. Among those providing structural data, more than 70% of YORA patients treated with biologic or targeted synthetic DMARDs exhibited no radiographic progression during follow-up. Although direct quantitative comparisons between younger and older populations were limited, younger adults generally demonstrated lower rates of structural progression suggesting a potential structural benefit associated with earlier intervention, although definitive conclusions are limited by heterogeneous imaging methods and relatively short follow-up durations.

Interpretation of these findings is constrained by heterogeneity in imaging modalities, scoring systems, and follow-up duration. Nevertheless, the available data suggests a trend toward improved structural preservation in younger patients, likely reflecting earlier therapeutic intervention and lower baseline joint damage.

### 3.4. Treatment Persistence and Drug Retention

Treatment persistence was reported across several cohort and registry studies and was consistently higher among younger patients. 12-month drug retention rates in YORA and younger adult subgroups frequently exceeded 70–80%, whereas discontinuation rates were higher in older-onset RA cohorts. When reasons for treatment discontinuation were reported, younger adults most discontinued therapy due to insufficient efficacy, while older patients more frequently discontinued because of adverse events or comorbidities.

Overall, treatment persistence appeared more favorable in younger RA populations, reflecting fewer comorbidities, improved tolerability, and greater capacity for sustained therapy.

### 3.5. Safety Outcomes

Safety outcomes were consistent with expected profiles of bDMARDs and tsDMARDs. The most common adverse events included upper respiratory tract infections, mild hepatic enzyme elevations, and hematologic abnormalities. Serious adverse events were infrequent among younger adults, occurring at lower rates than in older populations.

No included study identified age at onset as an independent risk factor for severe adverse events. Reports of malignancy, major cardiovascular events, or thromboembolic complications were rare across all age groups.

### 3.6. Summary of Findings

Overall, the available evidence indicates that younger adults with RA—including YORA and borderline younger-age subgroups—achieve better clinical outcomes, lower radiographic progression, higher treatment persistence, and fewer safety concerns compared with older-onset RA patients. However, heterogeneity in study definitions and outcome measures highlights the need for prospective studies explicitly addressing age-at-onset phenotypes.

## 4. Discussion

This systematic review synthesizes evidence from 16 studies evaluating clinical outcomes, radiographic progression, treatment persistence, and safety profiles of biologic and targeted synthetic DMARDs in young-onset rheumatoid arthritis (YORA) and younger adult RA subgroups. Although YORA represents a clinically meaningful phenotype, it remains underrepresented in the literature; only 6 studies explicitly defined age at onset, while 10 additional studies provided age-stratified outcomes relevant to younger adults. Despite this heterogeneity, several consistent themes emerged across the available evidence. It should be emphasized that age at disease onset is conceptually distinct from chronological age at assessment, as patients classified as older at enrollment may include individuals with long-standing young-onset disease.

Across studies, younger adults demonstrated more favorable clinical outcomes compared with older RA populations. Remission and response rates were consistently higher in YORA and younger-age subgroups, with DAS28 remission frequently reaching 40–60% at 6–12 months [43,44,45,46]. These findings align with previous observational data suggesting that younger patients exhibit a more vigorous immunologic response to therapy, earlier access to bDMARDs and tsDMARDs, fewer comorbidities, and greater adherence to treatment plans [47,48,49,50]. Importantly, no study identified diminished therapeutic effect in younger adults, supporting the concept that age at onset may reflect a biologically advantageous window for treatment responsiveness [51].

Radiographic outcomes, although reported in a smaller number of studies, further support this pattern. More than 70% of YORA patients treated with biologic or targeted therapies showed no structural progression [52,53,54], a proportion that appears higher than what is typically observed in older-onset RA. This likely reflects both earlier therapeutic intervention and the absence of long-standing joint damage at baseline. However, direct comparisons across age groups were limited by substantial heterogeneity in imaging methods and follow-up duration [55].

Treatment persistence also favored younger adults. Across multiple registries and cohort studies, persistence rates at 12 months commonly exceeded 70–80% among YORA and younger subgroups, whereas older patients exhibited higher discontinuation rates, largely attributable to comorbidities or adverse events [56,57,58,59]. These findings reinforce that the tolerability profile of bDMARDs and tsDMARDs is particularly favorable in younger RA populations and may contribute to sustained disease control over time [60].

Safety profiles were generally consistent across all studies, with younger adults demonstrating lower rates of adverse events. No unique safety signals specific to YORA were identified, and serious adverse events—including infections, cardiovascular events, and malignancies—remained infrequent [61,62,63,64]. These observations are further supported by large registry analyses and long-term extension studies of IL-6 inhibitors and JAK inhibitors, including baricitinib and filgotinib, which consistently confirm a favorable long-term safety profile under appropriate monitoring [65,66,67,68,69,70,71,72,73,74]. While long-term pharmacovigilance remains essential, current evidence supports the overall safety of biologic and targeted synthetic DMARDs in younger populations when appropriate monitoring is applied.

Despite these strengths, several limitations must be acknowledged. First, only 6 of the 16 included studies applied a strict age-at-onset definition of YORA, while the remaining studies relied on chronological age stratification [43,44,45]. This heterogeneity introduces unavoidable conceptual overlap, as older cohorts may include patients with long-standing young-onset disease. Second, the predominance of observational and registry-based studies limits causal inference and increases susceptibility to confounding by indication [52,53,54,55,56,57]. Third, outcome definitions and follow-up durations varied substantially across studies, precluding formal meta-analysis and limiting direct quantitative comparisons [58,59,60]. Finally, relatively few studies reported long-term outcomes beyond two years, restricting assessment of cumulative structural damage and lifetime safety in younger patients.

Importantly, young-onset rheumatoid arthritis should not be mixed with early rheumatoid arthritis defined by short disease duration. These entities represent distinct clinical constructs, and direct comparisons between them were beyond the scope of this review. Clarifying this distinction is essential for future research, as disease duration and age at onset may independently influence therapeutic trajectories and long-term outcomes.

Nevertheless, the findings of this review collectively highlight the importance of age at disease onset as a clinically relevant parameter in RA management. Younger adults—especially those with true YORA—appear to benefit substantially from early initiation of biologic or targeted therapies, achieving higher remission rates, better drug retention, and minimal radiographic progression [43,44,45,46,47,48,49,50,51,52,53,54]. As life expectancy and treatment options continue to expand, understanding the long-term trajectories of YORA becomes increasingly important for optimizing therapeutic strategies and minimizing cumulative disease burden over time [65].

Future research should prioritize prospective, age-stratified cohorts with standardized definitions of YORA and consistent outcome reporting. Longitudinal studies spanning multiple decades are needed to evaluate the durability of treatment responses, radiographic progression, and safety outcomes in this younger population [66,67]. Additionally, mechanistic studies exploring immunologic and genetic determinants of early-onset disease may clarify whether YORA represents a distinct biological phenotype within the RA spectrum [68,69].

## 5. Conclusions

This systematic review demonstrates that younger adults with rheumatoid arthritis, including patients with true young-onset disease, consistently achieve more favorable outcomes when treated with biologic and targeted synthetic DMARDs compared with older-onset populations. Across contemporary studies, younger patients showed higher remission rates, lower radiographic progression, superior treatment persistence, and a generally favorable safety profile, supporting the effectiveness of advanced therapies in this life-stage-specific population.

However, the limited number of studies explicitly defining young-onset rheumatoid arthritis by age at disease onset and the heterogeneity of age-based classifications highlight an important gap in the current evidence base. These findings underscore the need for prospective, age-at-onset-defined cohorts with standardized outcome measures and extended follow-up to better inform long-term therapeutic strategies.

Overall, age at disease onset emerges as a clinically relevant determinant of treatment trajectory and long-term prognosis in rheumatoid arthritis. Recognizing and systematically studying young-onset rheumatoid arthritis may enable more personalized, life-course-oriented management approaches aimed at sustained disease control and minimization of cumulative disease burden [72,73,74,75].

## Figures and Tables

**Table 1:** Summary of included studies reporting treatment outcomes in young-onset rheumatoid arthritis and younger adult rheumatoid arthritis subgroups.

Reference	Country/Setting	Study Design	Definition of Young Population	Total Study Population (*n*)	Relevant Younger Subgroup (*n*)	Therapy Evaluated	Key Outcomes Reported
Acuña-Rocha, 2024 [2]	Mexico	Systematic review & meta-analysis	YORA < 40 vs. EORA	11 studies	Not applicable	Mixed DMARDs	Higher baseline activity; earlier bDMARD initiation; higher remission vs. EORA
Ebina, 2023 [13]	Japan	Registry cohort	<40 subgroup	2.034	2.034	bDMARDs/JAK inhibitors	Higher drug retention; better sustained response in younger patients
Pérez, 2024 [6]	Argentina	Retrospective cohort	YORA < 40	211	211	csDMARDs/bDMARDs	Earlier bDMARD introduction; greater DAS28 improvement at 12 months
Miyamae, 2025 [7]	Japan	Claims-based cohort	Adolescents & young adults	5.321	5.321	TNFi, IL-6Ri, JAKi	Higher remission rates; faster escalation; lower discontinuation vs. older RA
Li, 2022 [9]	Canada	Registry cohort	Younger-onset < 55 (incl. < 40)	2.773	Not applicable	Standard RA therapy	Similar time to remission; younger patients escalated more often to advanced therapy
Semalulu, 2024 [40]	Canada	Transition cohort	JIA → adult RA (≤25 years)	≈300	≈300	bDMARDs	Persistent disease activity; high biologic use; phenotype overlaps with YORA
Cho, 2023 [5]	Korea	Prospective cohort	<45 subgroup	1.318	1.318	JAKi vs. bDMARDs	Greater clinical effectiveness and lower discontinuation with JAKi in younger adults
Cho, 2025 [14]	Korea	Prospective cohort	<45 subgroup	514	514	Tofacitinib vs. TNFi	Faster improvement and higher persistence in younger subgroup
Pappas, 2023 [15]	USA	Registry cohort	<45 subgroup	2.985	2.985	TNFi/JAKi	Faster achievement of low disease activity in younger RA patients
Lauper, 2022 [16]	19 countries	Multinational registry	Age-stratified (incl. < 40–45)	31,846	Not applicable	TNFi, IL-6Ri, JAKi, ABA	Higher remission and better retention in younger adults across all DMARDs
Lee, 2024 [18]	Korea	Registry cohort	Seropositive RA < 40	250	250	bDMARDs/tsDMARDs	Higher drug survival, especially with JAK inhibitors
Yamada, 2024 [21]	Japan	Prospective study	Younger vs. older RA	132	Not applicable	Abatacept	Reduced vascular progression and stable disease control in younger group
Bertsias, 2022 [22]	Greece	Cohort	Younger subset	70	Not applicable	Rituximab	Better treatment efficiency with fewer adverse events when used earlier
Eberhard, 2025 [41]	Sweden	Nationwide cohort	<45 subgroup	4.500	4.500	JAKi vs. bDMARDs	Greater pain reduction and better early response in younger adults
Tanaka, 2023 [28]	Japan	RCT extension	<45 subgroup	~100	~100	Filgotinib	Sustained clinical efficacy and stable safety profile in younger patients
Martinez-Molina, 2024 [42]	Spain	Real-world cohort	Younger adult JAKi users	402	402	JAK inhibitors	Younger age strongly predicted higher response and treatment persistence

YORA, young-onset rheumatoid arthritis; EORA, elderly-onset rheumatoid arthritis; bDMARDs, biologic disease-modifying antirheumatic drugs; tsDMARDs, targeted synthetic DMARDs; csDMARDs, conventional synthetic DMARDs; TNFi, tumor necrosis factor inhibitors; IL-6Ri, interleukin-6 receptor inhibitors; JAKi, Janus kinase inhibitors; ABA, abatacept; adolescent and young adult. Sample size refers to the total study population unless otherwise specified. Only age-stratified outcomes relevant to younger patients were extracted for the purpose of this review. Studies not explicitly reporting age at onset were included only if age-stratified outcomes for younger adult subgroups (<40–45 years) were available.

## Data Availability

The original contributions presented in this study are included in the article. Further inquiries can be directed to the corresponding author.

## Abbreviations

The following abbreviations are used in this manuscript:

ACR	American College of Rheumatology
bDMARDs	Biologic Disease-Modifying Antirheumatic Drugs
csDMARDs	Conventional Synthetic Disease-Modifying Antirheumatic Drugs
EORA	Elderly-Onset Rheumatoid Arthritis
EULAR	European Alliance of Associations for Rheumatology
HZ	Herpes Zoster
IL-6Ri	Interleukin-6 Receptor Inhibitors
JAKi	Janus Kinase Inhibitors
JIA	Juvenile Idiopathic Arthritis
PROs	Patient-Reported Outcomes
RA	Rheumatoid Arthritis
RCT	Randomized Controlled Trial
TNFi	Tumor Necrosis Factor Inhibitors
tsDMARDs	Targeted Synthetic Disease-Modifying Antirheumatic Drugs
VTE	Venous Thromboembolism
YORA	Young-Onset Rheumatoid Arthritis
